# Development and evaluation of a physical activity intervention informed by participatory research- a feasibility study

**DOI:** 10.1186/s12889-020-8202-2

**Published:** 2020-01-28

**Authors:** Rathi Ramji, Elisabeth Carlson, Anders Kottorp, Sergey Shleev, Eman Awad, Margareta Rämgård

**Affiliations:** 1 Department of Care Science, Faculty of Health and Society, Malmö University, Jan Waldenströms Gata 25, SE-20506 Malmö, Sweden; 2 Department of Biomedical Science, Faculty of Health and Society, Malmö University, Jan Waldenströms Gata 25, SE-20506 Malmö, Sweden

**Keywords:** Community-based participatory research, Action research, Health promotion, Health equity, Socially disadvantaged

## Abstract

**Background:**

Despite numerous interventions aiming to improve physical activity in socially disadvantaged populations, physical inactivity remains to be a rising challenge to public health globally, as well as, in Sweden. In an effort to address this challenge, a community-based participatory intervention was developed through active community engagement and implemented in a socially disadvantaged neighborhood in Sweden. The current study aims to present the development and initial evaluation of a participatory research driven physical activity intervention.

**Methods:**

Fifteen participants (11 females and 4 males) aged 17–59 years volunteered to participate in the physical activity intervention program. The intervention program was evaluated using a longitudinal mixed methods design measuring health impact changes over time through focus group discussions and quality of life surveys. Further additional biomedical health parameters such as levels of glycosylated hemoglobin, blood pressure, levels of oxygen saturation and body mass index were monitored before and after the intervention. Focus group data were analyzed using content analysis with an inductive approach. The pre-and post-test scores from the survey-based quality of life domains, as well as the health parameters were compared using non-parametric and parametric statistics.

**Results:**

Four themes emerged from the analysis of the focus group discussions including sense of fellowship, striving for inclusion and equity, changing the learner perspective and health beyond illness. The scores for the domains Physical Health, Psychological Health, Social Relationships and Health Satisfaction where significantly higher after participation in the physical activity intervention program compared to the pre-test scores (*p* < .05)s. There were however, no significant changes in the scores for the environmental domain and overall quality of life after intervention compared to that prior to intervention start. Overall, the biomedical health parameters remained stable within the normal ranges during intervention.

**Conclusion:**

The focus group discussions and results from the surveys and biomedical measures reveal important findings to understand and further develop the intervention program to promote health equity among citizens in disadvantaged areas. Evaluating the feasibility of such an intervention using multiple approaches contributes to effective implementation of it for larger communities in need.

## Background

There is countless evidences supporting that physical activity improves overall health and wellbeing [1–7]. The World Health Organization (WHO) defines physical activity as any form of movement of the body that leads to energy release owing to activation of skeletal muscles [1]. Physical activity also has a long-term effect in reducing the risk of chronic diseases [8–10]. Despite the known benefits of leading a physically active lifestyle, a high level of inactivity has been reported among adult population globally [11, 12]. The burden of chronic diseases has also been increasing worldwide and not the least in the developed world [13]. Studies show that physical inactivity contributes to a global burden of mortality as comparable with tobacco smoking [14]. Leading a physically active lifestyle may be influenced by factors such as physiological conditions, personal ability and psychological attributes [15]. Encouraging individuals and groups to be physically active has earlier been the focus of public health initiatives in a drive to prevent chronic diseases [16].

Sweden is one among the few nations in Europe meeting the World Health Organizations’ recommendation of performing physical activities at least 30 min daily. Nearly 65% of the Swedish population over the age of 18 years are reported to be physically active on a regular basis. However, there are reported differences in physical activity levels among subgroups of populations from disadvantaged backgrounds [14, 17–21]. Socially disadvantaged populations are diverse subgroups of the population who are heterogeneous with regard to their ethnicity, historical background, religion, culture, immigration status and practices related to health [22]. They may also differ based on income, employment, education, socioeconomic status and sociogeographical aspects as living in deprived neighborhoods compared to the majority population [14]. These subgroups commonly experience a higher risk of poverty, social exclusion, and discrimination. They are also exposed to several risky health behaviors owing to their poor living conditions [14, 18–21].

Several physical activity promotion campaigns addressing the whole population have proved unsuccessful since they often fail to reach out to these socially disadvantaged subgroups. Promoting healthy eating and exercise with consideration to the social dimension provides a better opportunity to initiate healthy lifestyle [23]. Therefore, an alternative approach in public health efforts aiming at health promotion may be necessary to understand the impact of individual action together with the context, while designing preventive measures against risky health behaviors in socially disadvantaged populations [16]. Taking into consideration specific social requirements and increasing community engagement to improve health, is an alternative paradigm for health and social research, namely the Community-based Participatory Research (CBPR) [24]. This form of research involves close collaboration between different stake holders such as academic partners and the community in order to identify communities priorities. The partnership between community members, who have direct knowledge of local circumstances and the academicians, is central in developing successful health interventions. This partnership identifies health issues that are of concern to the community and addresses it through research and action. CBPR helps generate relevant evidence through integrally involving community members throughout the research process [25]. This research approach is considered appropriate particularly since the communities themselves are involved in designing interventions that focus on the interlink between health behaviors and the social determinants of health. Success in these efforts is achieved by working with the communities to not only understand their needs and developing an intervention but by further empowering them, to address multiple barriers to health within their socio-cultural context [26]. CBPR allows working collaboratively with the community members through building trust, and sharing power throughout the different stages of the research [25]. The process of building trust between the community and the different actors, paves way to understand the multiple and complex factors associated with health of citizens in the community. Furthermore, it facilitates the assessment of behavioral and environmental factors that influence health and well-being and thereby guides the development of successful health promoting interventions [25, 27]. CBPR has the potential to not only promote health but also informs the need for changes in practice and policies. Above all, CBPR aims to build community capacities to ensure sustainable change [25].

There are a few international studies on physical activity interventions guided by a CBPR approach [28–31]. Moreover, studies evaluating CBPR based intervention using multiple health evaluation methods longitudinally [26] are sparse. This study aims to present the development and initial evaluation of a participatory research driven physical activity intervention built by an established CBPR partnership with communities from socially disadvantaged neighborhoods in Sweden.

## Methods

### Context

The current study was part of a larger initiative developing and studying health-promoting activities informed by community-based participatory research in a socially disadvantaged neighborhood in a city located in the South of Sweden. There is a large proportion of non-Swedish speaking population residing in this locality. The locality has been considered one of the twelve most socially deprived areas in the country [32]. The municipality in this region initiated a collaborative program that [33] emphasized the need for sustained efforts to improve this part of the city. There is a high rate of unemployment, crime, low education levels and poor health among residents. Previous studies in the city have also identified high incidence of risky health behaviors among citizens in this neighborhood [20, 21].

### Development of interventions and health promotion resources

The physical activity intervention being described in the current study is part of a larger project [34]. The larger project began with future workshops [35] where 150 citizens of different age groups and genders from the geographical community participated. The discussion within these workshops aimed at identifying initiatives to promote health, well-being and quality of life of the community based on their perceived needs. The discussions revealed that sedentary lifestyles or physical inactivity, poor oral health among children, mental health problems and reduced access to coping strategies are factors affecting the self-perceived health of this community. In addition, the citizens perceived a lack of access to health care, specifically evaluating and monitoring their health at a low cost, understanding their own health status, and lack of access to information on healthy diet in their area. The citizens perceived the need for activities free of cost in the area and held during the day. The citizens of the locality together with different stakeholders from private, public, non-profit sectors and academic partners created a CBPR model influenced by a previously tested model [26, 36] in a similar context for strategic planning of collaboration and development of health interventions focusing on key factors such as empowerment and participation at all stages including planning, implementation and evaluation.

The health promotional activities focused on five problems areas including (a) physical activity, (b) mental health, (c) self-care, (d) oral health and (e) health literacy.

The health promotional activities are coordinated by representatives from the locality, often known as community health workers or multicultural health brokers or gatekeepers in an international context [37], but in Swedish context they were known as *health promoters.* The health promoters were among the citizens who were part of the initial future workshop who expressed their interest in assisting the research team and being change agents for their community. These health promoters were instrumental as facilitators for participant recruitment, language interpretation and above all informing the research partners about the nuances and cultural sensitivities within the community to be considered when approaching individuals for various activities. In addition, they have in-depth knowledge and experience of the issues faced by these communities in accessing health and services, and are likely to offer culturally appropriate support to the research project. Previous research indicates that individuals who share a similar background with the target groups are better able to respond to the health needs of these subgroups [37].

This was the first stage in the development of the intervention program being evaluated as a part of the current study.

### Development of the physical activity program

After the strategic planning and development of CBPR model, about 70 members from the community, together with the health promoters participated in a new workshop to develop a socioculturally appropriate physical activity program. The academic partners together with one of the health promoters who was a fitness specialist facilitated this workshop together with different stakeholders. Dialogues were held within the community regarding expectations from the physical activity program, as well as, the best ways to deliver it. Discussions were also extended to explore the cultural factors that may influence participation in the physical activity program. The main points from the discussions were categorized together with the community members to identify relevant action points. Community members suggested that an ideal physical activity program must include exercise and training that is tailored to individual capabilities. They wanted one of the health promoters to be the personal trainer. Community members wanted training to be related to the daily activities they perform rather than working on devices or machines so they can learn to handle their body in the right way using tools that are easily available. Participants also preferred having gender specific groups since their culture did not permit women training with other men. Participants wanted to be counselled about healthy diet and healthy mind, as they frequently experienced lack of motivation when training alone. In addition to physical activity, participants also wanted to follow the effect of physical activity on their health.

A unique fitness program was framed by the health promoters based on the input from the community members. The structure of the program was presented to the community members who participated in the development workshop for feedback and approval. The main purpose of this fitness program was to build community and diversity together for a fair and equal health and fitness culture among the citizens in the neighborhood based on the perceived needs. The fitness program focused on facilitating a gradual change in the citizens’ lifestyle, from simply moving the body and eventually to learning training methods based on their own abilities. By doing so, one develops healthy routines that in turn creates self-awareness of their own body and therefore self-esteem and self-confidence can grow. The training as a part of this program aimed to promote change without distinction by age, language, socioeconomic status or cultural identities. The physical activity program was also designed such that it could be offered free of cost and thus accessible to those with a disadvantaged background. Through integrating people of different ages and with different cultural backgrounds, an additional incentive is that the program promotes increased integration among citizens in the neighborhood. Participants in this program gain social support from within the group of actors and other citizens, which motivated them to make lifestyle changes, based on their own perceived living conditions. Aside from training participants to perform physical activity, this program also educated participants to become future trainers. As future trainers, these individuals can train their acquaintances in the neighborhood, thereby spreading the fitness culture to more members in the community. A prior study has also identified the usefulness of the train the trainer method in spreading interventions to larger communities [38].

### Participants

Health promoters approached 20 individuals from the neighborhood who were also part of the physical activity program development workshop to participate in the physical activity program and the research study through face-to-face interactions, as well as, using messages and flyers. It was only noted, if the participants were in good health with the ability to perform physical activity and free from medical conditions that might hinder participation. Participant were not asked about prior training experiences or ranked using standard scales, rather included as health seekers who volunteered to participate. Five participants dropped out due to the inability to understand the design of the intervention and lack of time. Participants who mentioned lack of time expressed that they really wanted to participate however they were unable to since the time scheduled for the physical activity program did not match with their daily routines including attending language classes, or caring for their children or a family member. The health promoters and the participants in this study were ordinary citizens from the neighborhood and did not hold any specific positions within the community and were not politically or otherwise involved outside of the community.

The participants were organized into two training groups with eight to ten persons in each of the two groups. Each group met once a week for about 2 hours over a period of 3 months. During each session, participants performed physical activities outdoors, as well as, indoors together with the health promoters with periodic breaks between the training spurts, which lasted for about 60 min. In addition to training, participants were also involved in a dialogue about eating healthy and the relationship between diet and physical activity. At the start and end of each session, participants also sat in circles to reflect and share their experience with one and other as well ask questions.

### Study design

The current study used a concurrent mixed method evaluation model where both qualitative and quantitative data were gathered over time. The quantitative method was quasi-experimental assessing the differences within subjects utilizing a pre-test prior to intervention start and a posttest directly after the end of the intervention. Qualitative data was also collected at similar two time points, pre- and post-intervention.

### Focus group interviews

Focus group interviews were conducted in two separate groups with six to seven participants in each group before starting the physical activity intervention program (t1), and at the end of the intervention program (t2). Each interview lasted for about 2 hours and discussions proceeded until data saturation was achieved. The questions posed in the focus groups aimed at gaining a deeper understanding of the participants’ perceptions of participation in a process where several different actors work together. The focus groups were conducted using an interview guide based on the CBPR model [25]. The model relates to how participants perceive participation in the group they belong to and how they perceive and think about the collaboration with the participating partners, including the health promoter who leads the physical activity program and university employees who participate in the evaluation of the group. Since the program is designed as an intervention based on the participants’ own needs, it was important to ensure that all participants in the program experienced compliance with actors and other participants. Minimizing power relations was necessary so individuals could develop themselves, with the support of the program, to change their health.

### Surveys

Participants were asked to respond to the World Health Organization Quality of Life-BRIEF survey (WHOQOL-BREF) [39]. WHOQOL-BREF was chosen as appropriate for this study as it examines the perceptions of individuals in relation to their culture and value systems, as well as, their personal goals and standards. The validity and reliability of the instrument has been tested in 15 field centers around the world and the instrument is available in 20 different languages including Swedish [39] and Arabic [40]. The instrument includes 24 items, each scored from 1 to 5 measuring quality of life scores across four domains. Physical domain is the first of it with seven items; examples include questions on pain, fatigue, and use of medication. In the psychological domain are six items including questions on stress, anxiety and concentration abilities. The social relationships domain is the third with three items focusing on social support and relationships. Finally, the environmental domain, which included eight items focusing on physical environment and access to health care. In addition to the four domains, two questions measuring general quality of life and health satisfaction were also included.

A raw score of each domain was calculated and transferred into range between 0 and 100 for compatibility with the scores used in World Health Organization Quality of Life-100 item survey (WHOQOL-100) as directed by the standard guidelines provided by the WHO. Higher scores denote higher quality of life [39].

In addition to this instrument, additional information including age, gender, mother tongue, educational qualifications, current employment status, diet and physical activity levels were included. Further, information related to socioeconomic status including household income was also gathered.

### Health tests

Given the known effect of physical activity on the incidence of chronic disease in adults [9, 13], there is a need to assess of metabolic risk markers of chronic disease such as body mass index, blood pressure, blood sugar and level of oxygen saturation in blood, as a part of evaluating health effects of the physical activity intervention [41, 42]. These assessments were also included since they were in line with participants need as indicated by them during the initial workshops.

Height in centimeters was measured with a length scale MZ10032 from ADE (Hamburg, Germany). The information on height in centimeters was manually entered into the Tanita Model MC780MA (Tokyo, Japan) which was primarily used in the current study to assess the weight in kilograms as well as to calculate the BMI using Eq. 1:

1 $$ \boldsymbol{Body}\ \boldsymbol{Mass}\ \boldsymbol{Index}=\frac{\boldsymbol{Body}\ \boldsymbol{Weight}\ \left(\boldsymbol{kg}\right)}{\boldsymbol{Body}\ \boldsymbol{Heigh}{\boldsymbol{x}}^{\mathbf{2}}}\kern24.75em $$

Blood pressure was measured using an iHealth Sense Wireless Wrist monitor from iHealth (California, USA). The device measured systolic and diastolic blood pressure (mmHg), as well as, heart rate (bpm). Level of oxygen saturation (%) was measured using an Angioscan 01 from Angioscan Electronics (Moscow, Russia). Blood was collected by capillary blood sampling to investigate the level of glycosylated hemoglobin in blood (%) as an indicator for long-term glycemic control using a HemoCue HbA1c 501 System from HemoCue (Ängelholm, Sweden). Assessments of the health parameters were performed in comparison to their reference intervals. The reference intervals for oxygen saturation and for body mass index (BMI) were set to be 95–99% [43] and 18.5–25.0 kg/m^2^ [44], respectively. Systolic and diastolic pressure was assessed using reference intervals 100–140 mmHg and 60–90 mmHg [45], respectively. The reference interval for heart rate at rest was considered to be 50–90 beats/min [46]. As for glycosylated hemoglobin, the cut-off value for healthy individuals was assumed to be 6.5% [47].

The health tests were performed at two time points once before the program start (t1) to ensure stability between the test parameters, and the second measure was made after the completion of the physical activity program (t2).

### Analysis

#### Qualitative analysis

The focus group discussions were recorded and transcribed verbatim. Interviews were conducted primarily in Swedish since most of the participants spoke Swedish sufficiently despite having different cultural backgrounds. The analysis aimed at capturing change in perceptions of participants before and after participation in the intervention. An inductive content analysis of the transcribed interviews was conducted. Unlike other qualitative analytical techniques, an inductive approach is usually used to uncover key categories that are relevant to the research objectives [48]. After verbatim transcription of the focus group discussions, the transcripts were thoroughly examined by two members of the research team to identify codes for each response through repeated reading. Similar codes were then grouped together to more general themes. The process was repeated a few times until concrete themes emerged and no further themes were identified. The researchers worked on the original transcripts until no new insights were identified. A joint list of themes were finalized after discussions between the first two researchers. A third researcher also a member of the research team, independently worked on the transcripts following similar guidelines followed by her colleagues. The third researcher then reviewed results from the analysis made by the first two researchers, and validated their findings. Thus, triangulation was achieved.

To ensure trustworthiness and address issues such as reflexivity, the research team involved in analyzing the data attempted to set aside their own pre-understanding of the social setting and contextual factors associated with the population and locality. Furthermore, reflexivity was also addressed through meticulously planned methodological consideration and analytical procedures including peer debriefing and triangulation through inclusion of several persons in both the focus groups (as observers), as well as, in transcript analysis [49, 50].

#### Quantitative analysis

Since the current study is a feasibility study, and that similar studies with this population have not been made earlier data necessary for a power analysis is not available. Comparison of the scores of quality of life domains before and after the fitness program was done using Wilcoxon’s Signed Rank Test models, due to small sample size and ordinal data. Analyses focused on group level over time to identify patterns in changes, but we also monitored changes on individual levels. Statistical significance was set at a *p* value of < 0.05. Effect sizes were computed and interpreted using the following effect sizes references, *d* = 0.2 or lower was considered small, *d* = 0.5 as medium, and *d* = 0.8 as large [51].

For the health parameters, tests of normality were initially performed to find out which type of statistical analysis should be used in determining statistically significant changes between the first and last measurements (t1 and t2 values). Using the Shapiro-Wilk test with a significant level of *p* < 0.05 it was found that all data were normally distributed apart from BMI [52]. The paired samples *t*-test was therefore used for all health parameters, except BMI due to its assumption of normality within the data set. Indeed, for BMI the Wilcoxon Signed Rank test was used instead of the paired samples *t*-test [53]. The effect size in the Wilcoxon Signed Rank test was calculated using Eq. 2:

2 $$ \boldsymbol{r}=\frac{\boldsymbol{z}}{\sqrt{\boldsymbol{N}}} $$

Triangulation of methods was performed to additionally validate the results through a comparison of questionnaire results with that of the focus groups interviews.

### Ethical considerations

All participants were initially informed that they will participate in a research process with their participation in the physical activity program. They were also informed that the purpose of the research is to investigate the impact of the fitness program on the health and well-being of the participants and not on individual performance. Participation was voluntary and participants were informed that could leave the study at any time. Written information on the research process was also provided and participants were asked to sign an informed consent form. All material collected were marked by code, and are kept confidential and shall be accessed only by the research team members. Prior to initiation, the Regional Ethical Committee in Lund approved this study (DNR 2018/384).

## Results

A total of 15 participants (11 females and 4 males), aged 17–59 years participated in this feasibility study. There was over 95% attendance rate in all sessions. Participants who missed a training pass often did so because of caring for their sick child or if they were unwell themselves. Participants were predominantly were of Middle Eastern origin with a few Europeans, and Asians. Table 1 includes a description of the participant’s demographics. Although all the fifteen participants considered themselves active through performing daily household chores they had not been involved in physical activity or training ahead of their participation.

**Table 1:** Characteristics of participants

Characteristics	Participants (*n* = 15)
Age	
Range (md)	17–59 (32)
Gender	
Women (%)	11 (73%)
Men (%)	4 (27%)
Ethnicity	
Asian	3 (20%)
European	2 (13%)
Middle Eastern	10 (67%)
Educational Qualification	
University education (≥ 3 years)	4 (26%)
University education (< 3 years)	1 (7%)
Adult education at high school level	1 (7%)
High school (3–4 years)	3 (20%)
High school (2 years)	3 (20%)
Elementary school	3 (20%)
Occupational status	
Employed	5 (33%)
Self-Employed	1 (7%)
Parental leave	6 (40%)
Studying/Internship	1 (7%)
Unemployed	2 (13%)

### Qualitative findings

#### Process evaluation of the physical activity intervention

On exploring, the focus group interviews concerning participant’s views on the physical activity intervention revealed four main themes including sense of fellowship, striving for inclusion and equity, changing the learner perspective and health beyond illness.

#### Sense of fellowship

Sense of fellowship in this study was about how individuals developed interest over time to be physically active and healthy through being inspired by others in the group. Participants, who were initially disinterested and joined the intervention program for the sake of their friends, started liking the activity within the group eventually. They believed that they gained more while being in a group and desired to be role models to others.

Before participating in the physical activity intervention, participants perceived lack of motivation to train by themselves. They frequently chose not to train if they were to train alone. They needed a companion who could drive them to train and even participate in the intervention program.

*“When I enrolled myself, I had no desire to come and train, whatever type of training it be, I came because my friend persuaded me to.”(Pre-interview, female)*

However, when participants started training they started to develop a sense of togetherness with other participants in the group, which positively influenced them to continue training. Participants felt committed to the group and were determined not to miss training passes due to respect for the fellow participants.

*“Tomorrow, I have a scheduled training pass, Ohh it is tomorrow.. I feel tired and believe I cannot manage. But, no I cannot do that, I must respect. I must respect others in the group. We must keep our word, if we promise.” (Post- interview, female)*

On starting to train, participants started to gain interest in training especially since they were training in a group. They usually felt demotivated when they had to train all by themselves.

*“It was nice training with the group. Because we laugh, we talk, it's fun for us, so. When we train by ourselves, it's boring. It's not possible" (Post- interview, female)*

Participation in the program also made participants glad as it was also about meeting new people and getting to know them, thus expanding their social network in the locality. Becoming familiar with more persons increased their sense of safety in the locality.

*“Through training in these new groups, with new people, gives a sense of security in the locality. We get to know new faces” (Post- interview, female)*

Participants also expressed their desire to share the knowledge they had received with others. However, they believed that taking care of self, practicing a healthy lifestyle, training and showing others through action could help spread the intervention to others more effectively than merely sharing experiences verbally.

*“Taking care of oneself, like to live healthy, train and we try to show it to others by being a role model. Thus, for example, people are watching me, they see that I have lost weight in the last month. I said I had been in training, and I told them what we were doing during this month. "(Post- interview, female)*

#### Striving for inclusion and equity

Striving for inclusion is a voluntary action taken to meet the needs of different people. It includes creating environments or situations where everyone feels included and welcome. Through the discussions with participants emerged a general sense of isolation among citizens in this locality. The citizens, predominately women restricted themselves to their homes, and often avoided external contact. Many were not even aware of the activities and possibilities available in the locality. This was primarily owing to their family situation, psychosocial conditions and the lack of interest. There was a general lack of motivation particularly to get involved with physical activity. They were dependent on the company of their friends or family members to go out of their homes.

Participants described lack of access to health related activities for women in the locality at the beginning of the intervention, but with eventual participation, they started to believe that they had the potential to drive change. Towards the end of their participation in the intervention participants realized that they can create a positive atmosphere which welcomes especially those that were excluded.

Women from Middle Eastern culture do not train in public places such as recreational facilities and sport gyms due to the presence of men. Participants perceived that there was a lack of access to recreational activities, specially directed to women in the close proximity. Women in the locality felt excluded since they were not able to perform physical activity even if they desired to do so. Some women had disabilities preventing them from going to the sport centers or gym even if they had exclusive training times for women. They really wanted to train but often excluded since there were no activities adapted to needs and capabilities of women.

*“ There is no access to training or physical activity targeting woman in the locality earlier. There was a need for training or swimming or something else that is adapted and is only for women. They just sit here and cook or sew or do something. But do nothing special like exercise. ” (Pre- interview, female)*

When participants started attending the intervention program, they came to understand that the responsibility to create an open environment was within every individual. In the current study, most of the women participants did not mind participating in the intervention program together with men. Some women who refused to perform certain activities within the intervention program in front of men, made notes and videos of training and tried it at home instead. They also had the opportunity to film themselves and take help from the trainer. Participants believed that they could also be part of building social health where everybody cares for the other.

*“I believe that it is we who can create this a welcoming environment for people. It is only we who can open the doors for everyone. We need more of this "community care" we can together build social health wherever we are. "(Pre- interview, female)*

After participation in the intervention program, participants felt that they could spread positive energy to others that in turn makes others in the locality feel more welcome to participate.

*"It's good that there is someone who can help others. That they feel welcome to train together in a group. To give them also the energy and chance to train now, even if they can't do it all by themselves. "(Post- interview, female)*

#### Changing the learner perspective

Changing the learner’s perspective was about strengthening individual knowledge and capabilities in collaboration with others such as a physical instructor or members in a group. Individual participants moved from stage of seeking knowledge to becoming coaches themselves during the process of participation in the intervention program.

Prior to the intervention start, the participants believed that having a personal instructor was ideal since it would help them create an individually adapted training regime however, they could not afford such a service, as it was expensive. Through the intervention program participants felt that they had, the opportunity to learn from a person with such a knowledge. The coach who was also a health promoter was not an external person but was also from the locality and he shared knowledge concerning the challenges and needs of the citizens in the locality. Participants believed that they could learn better under the guidance of the coach about what was most suitable for them as individuals.

*“I want to start by learning what to do, to make my body feel better, learn about health, food and training. For example, which training is better for me. I want to do something that is good. He can teach me, and he knows which is best, so one can feel good and be healthy ” (Pre- interview, female)*

Participants were aware that physical activity and food were interrelated determinants of healthy body functioning. Therefore, they were curious to learn how to work with both so the body is in a state of balance.

*“I think food and training are completely for the body, because if you train and eat sweets and fatty food, it does not work. And it’s the same, if you eat healthy and don’t move your body. One must learn how to balance with both.” (Post- interview, female)*

After starting to train with the help of the instructor and learning for themselves, participants went on to spread their knowledge to their family members. Participants aspired being instructors in future and hence tried sharing what they learnt from their own participation in the program with their own families.

*“I have started to train at home with my mother, father and my siblings. I do the same things that we do here, as to talk and train with them. I try to teach them what I myself have learned here.” (Post- interview, female)*

At the end of the intervention program participants expressed their desire to be future ambassadors for the program and spread it to more people from the location.

*“I think , I would like to be a coach like a training group leader and show others what I have learnt.” (Post- interview, female)*

#### Health beyond illness

There was a general lack of trust in the health care system among citizens in the locality which was reported ahead of participation. The citizens in the neighborhood expressed that the health system was not similar to that in their home country. They also believed that they did not get the help they expect when they approached the primary care centers and thus did not trust the system. Participants perceived that patients had to wait for long to meet a doctor. Even if they did meet a doctor for a specific problem, they were not provided diagnosis and treatment in time. Most patients are offered paracetamol and asked to wait for prolonged periods before being diagnosed and actual treatment is provided. They felt uninformed about their health and the different health parameters that can be monitored early in time to understand the risk of getting exposed to diseases such as diabetes and other cardiovascular disorders.

After participation in the physical activity program participants developed an understanding that healthy lifestyle and training contributes to better physical and mental health and thereby a better quality of life.

*“I feel healthy and brisk and there is a difference since the beginning to now. I think of my body, and what exercise I have to do, which food is better, which is not so good. Exercise makes me feel good and I started to feel that my body works, that I feel good mentally, it is beyond just health .. I am happy.” (Post- interview, female)*

Participants were very excited about the health tests since there was a lack of trust in the health system in general. Participants appreciated the health test even better since they had been offered the time and contact when being tested that they had the opportunity to learn the significance of different parameters tested.

*“I have no confidence in the health care system, so I felt it was so luxurious to do tests. There came a guy there and we asked about everything he tested, what does that mean? What does this mean? How can I do that? Can I do something about it? Then, that you were told that you were like 20, 25, it just did something even better for us.” (Post- interview, female)*

Some participants were curious about their results after participation in the intervention program, and were extremely disappointed if the results were not as they expected. It was perceived that they were intending to quit the program if their health status did not change.

*“Some in the group are eagerly awaiting the results of the test. They think about it all the time. If results are not as expected they are disappointed. It could make them give up, and not continue. ” (Post- interview, female)*

Some others in the group were careful in not drawing motivation for participation through the results from the health tests. Participants believed that they must focus on their own health and lifestyle through being physically active and eating a healthy diet. By doing so, they believed that the changes in health status might eventually happen.

*“I don't focus much on the testing. I focus on my health and on my lifestyle, how it should be. With diet, exercise and one can improve one’s own health by themselves. ” (Post- interview, female)*

### Quantitative findings

The scores for the domains Physical Health, Psychological Health, Social Relationships and Health Satisfaction were significantly higher/better after participation in the 12-week physical activity intervention program compared to the pre-test scores (see Table 2), with a moderate effect size in all domains. However, the tests did not elicit a statistically significant change in scores representing the Environmental domain, as well as, self- rated quality of life after participation in the 12- week physical activity intervention program. Table 3 includes a description of results from the Wilcoxon’s signed-rank test.

**Table 2:** Descriptive characteristics of quality of life scores, and health parameters before and after intervention

Domain/Health Parameter	t1-Mean (SD)	t2-Mean (SD)
Physical Domain	58.81 (21.6)	69.95 (19.8)
Psychological Domain	56.94 (20.3)	66.08 (19.1)
Social Relationships Domain	64.44 (15.8)	71.66 (15.3)
Environmental Domain	57.71 (15.8)	59.58 (14.7)
Quality of Life	3.33 (1.2)	4.13 (0.5)
Health Satisfaction	3.13 (1.1)	3.93 (0.7)
Body Mass Index (kg/m^2^)	25.17 (5.9)	25,03 (5.8)
Systolic Blood Pressure (mmHg)	123.87 (12)	119.27 (16.4)
Diastolic Blood Pressure (mmHg)	78.47 (7.4)	74.6(12)
Heart Rate at Rest (BPM)	73.2 (7.7)	72.93 (6.4)
Blood Oxygen Saturation (%)	96.23 (1.2)	96.31 (1.0)
Glycosylated Hemoglobin (%)	6.19 (1.3)	5.93 (0.4)

t1- pre-test; t2- post-test

**Table 3:** Wilcoxon signed rank test comparing quality of life domain scores and health tests before and after the physical activity intervention

Comparison of QOL Domains/Health test	Mean difference (SD)	z	*p*-value	Effect size
Physical Health Domain t_2_-Physical Health Domain t_1_	7.14(8.5)	-2,59	0.009*	0.47
Psychological Health Domain t_2_-Psychological Health Domain t_1_	9.14(12.0)	−2.77	0.006*	0.50
Social Relationships Domain t_2_-Social Relationships Domain t_1_	7.22(12.5)	−2.07	0.039*	0.38
Environmental Domain t_2_-Environmental Domain t_1_	1.87(9.86)	−0.75	0.450	0.14
Self-Rated QOL t_2_- Self-Rated QOL t_1_	0.80(1.4)	−1.92	0.055	0.35
Satisfaction with Health t_2_ – Satisfaction with Health t_1_	0.80(0.9)	−2.52	0.012*	0.46

*QOL* Quality of life; t_1_-pre-test; t_2_- post-test; * *p*-value < 0.05

### Health tests

The Wilcoxon Signed Rank test and the paired samples *t*-test showed that there was no statistically significant mean difference between the values of biomedical parameters, viz. BMI, heart rate at rest, systolic and diastolic pressure, as well as, glycosylated hemoglobin, measured before and after the physical activity intervention on a group level (Tables 4 and 5).

**Table 4:** Wilcoxon Signed Rank test comparing body mass indexes before and after the physical activity intervention

Comparison of Health test	Mean difference	z	*p*-value	Effect size	Number of measurements
Body mass index t2- Body mass index t1 (kg/m^2^)	0.14	−0.06	0.95	−0.33	15

t_1_-pre-test; t_2_- post-test

**Table 5:** Paired samples *t*-test comparing biomedical parameters before and after the physical activity intervention

Comparison of Health test	Mean difference	t	*p*-value	Number of measurements
Systolic pressure t_2_ - Systolic pressure t_1_ (mmHg)	4.60	0.99	0.34	15
Diastolic pressure t_2_ - Diastolic pressure t_1_ (mmHg)	3.87	1.19	0.25	15
Heart rate at rest t_2_ - Heart rate at rest t_1_ (bpm)	0.27	0.18	0.86	15
Blood oxygen saturation t_2_ - Blood oxygen saturation t_1_ (%)	−0.08	−0.32	0.75	13
Glycosylated hemoglobin t_2_ - Glycosylated hemoglobin t_1_ (%)	0.25	0.84	0.42	13

t_1_-pre-test; t_2_- post-test; Due to some technical difficulties data for two volunteers were missing, when it comes to blood oxygen saturation t_2_ and glycosylated hemoglobin t_2_

## Discussion

This feasibility study presents findings from the evaluation of a physical activity intervention informed by community-based participatory research aimed at communities residing in a socially deprived locality in Southern Sweden. This study primarily aimed at, presenting the development and initial evaluation of a participatory research driven physical activity intervention through triangulation of data, collected using different methodologies. In an attempt of doing so, the study also provided insights on both challenges as well the aspects contributing to the successful implementation of this evaluation approach to examine a complex intervention.

The current study elucidated the importance of the group process in the successful implementation of a physical activity intervention. Group activities promoted collaborative actions creating an environment for positive interpersonal influence that lead to commitment, which in turn influenced behavioral change. Development of group skills strengthened individuals’ faith in their own abilities and boosted their self-confidence, which successively led to personal empowerment. This is in line with previous studies describing social support as an important determinant of successful implementation of health promotional intervention [54–57]. However, in the current study participants became empowered through social interaction with other participants in the intervention program, which gave them the confidence to spread their knowledge to others thereby creating a wider social network bounded by common goals such as physical activity and health. This was in line with the process of empowerment explained in a previous study [58], specifying three central components including that empowerment must lead to an increased control of one’s own health which further leads to an increased ability to control their life and thirdly it should increase the ability to change the world . The process is reflected in our current study through participants perceiving a change in their own health followed by the emergence of the thought that health was beyond being disease free, which gives the feeling that participants achieved a higher control over their life through their participation. Through being coaches at the tertiary level, the participants were inclined towards changing the world.

In addition, participants in this study were unacquainted to each other ahead of participation however, they developed a sense of trust among each other and thereby an increased sense of security in the locality in the group during the course of 12 weeks. This was in contrast to previous studies describing the mechanism of social support in promoting health where social support was derived primarily from family members and friends [59–61]. The current study shows that empowerment can arise while meeting with other unknown people especially when they are bound by common goals. This helps them as individuals to take control of factors that improve their quality of life especially with health as focus.

In line with previous studies [62–64], participants in this study, specifically women with Middle Eastern backgrounds perceived an unavailability of activities that were exclusive for women ahead of participating in the intervention program. Through participation in the current intervention program women from the community felt motivated to become role models and engage themselves in creating new and meaningful initiatives where secluded groups within the community in particular women are welcomed. Previous studies have recommended the need for involving women representatives from the community in designing such activities to increase social inclusion [64], but no such interventions have been implemented and evaluated prior to the current study.

Participants in this study became more self-sufficient through participating in the intervention program. Although they started with being curious to acquire new knowledge from others and thus were dependent on the coach or other participants in the group, they eventually improved over time to the extent that they were confident of becoming coaches themselves and sharing their knowledge with others in their community. The application of the train-the-trainer educational model together with the physical activity intervention in the current study made participants confident in their capabilities to spread the intervention to other members of the community. Train-the-trainer model has earlier also been proven successful in enhancing public health preparedness in particularly in disadvantaged communities [38, 65, 66]. There has however been limited research on the impact of CBPR informed invention in Middle Eastern migrant population, particularly women. However, existing literature has recognized the value of the participatory process in adequate adoption and spread of interventions targeting lifestyle modifications [66].

In an attempt to understand the impact of the intervention the study also shed light to the barriers experienced by participants that may influence the intervention. Participants related to the lack of motivation to train, lack of access to culturally sensitive physically activity programs primarily for woman as barriers to being physically active ahead of the intervention. After participation in the intervention, however they also discussed how the intervention help them to overcome some of the barriers. Empowering women and encouraging them to participate in intervention programs focusing on building trust among community members was seen as way to overcome these barriers [67].

Among the four domains and two global items, measuring quality of life assessed before and after participation in the intervention program there was a significant improvement in physical health, psychological health, social relationships, as well as, perceived satisfaction in health among participants in this study. These results are in line with results from previous studies assessing the health effect after participation in behavioral interventions [68–71], however these studies targeted subgroups of population such as older adults or population cohorts with a particular disease or recovering from illnesses. Interestingly, there were no changes identified in the environmental domain before and after participation in the intervention. An explanation to this could be that the components assessed within this domain include higher-level factors such as policy changes, accessibility to health care, financial situation and safety. These factors will not be directly impacted by a short-term physical activity intervention. These results were in line with previous studies involving older adults [63] unlike the current study, which included a subgroup of population in different age groups.

Interestingly the results from the quantitative surveys were very consistent with that of the findings that emerged from the focus group interviews, supporting triangulation of the findings. Participants experienced better health and wellbeing overall with improved physical and mental health. The aspect of being together and the effect of working in networks through bonding and bridging have emerged both from the quantitative, as well as, qualitative results. However, it was also evident that participants remained unsatisfied about the access to health care throughout their time in the intervention program, which reflected on the environmental aspects of the quality of life assessments. The triangulation of the results from the qualitative and quantitative studies, have reached similar conclusions, which not only indicates the possible validity of the results, but also provides a deeper understanding of the underlying mechanisms behind the possible success of the intervention.

Participation and trust emerged as key aspects guiding the success of the intervention in the current study. In contrast to interventions implemented at a population level, Transformative CBPR interventions are grounded on the principles of CBPR which also emphasizes the pivotal role played by participation (defined as a process of organizing to achieve meaningful social change), in influencing health outcomes at a community level [26]. Through merely participating in the intervention program participants in the study experienced changes not only in their lifestyle and health but also their social life and existence in the locality.

The initial evaluation of a participatory research driven physical activity intervention was demonstrated to be feasible in that it was highly acceptable to participants, while also elucidating significant improvement in self-reported health and quality of life, together with individual improvements in some parameters, e.g., glycosylated hemoglobin. One should note that many participants in this sample demonstrated biomedical measures within the reference intervals already at the pretest evaluation, which indicates that no major changes were expected in relation to the physical activity intervention. The triangulation of methods used for evaluation of the interventions indicates that using multiple perspectives and indicators of health is important to include as outcomes in order to determine the complex effects of a CBPR based health intervention as in this study.

On the one hand, positive outcomes mirror previously published studies in similar groups in Sweden, as well as, elsewhere in the United States [57, 72]. On the other hand, the current mixed method evaluation study differs in principle from previous investigations. Specifically, this CBPR informed intervention has not only proven to be vital in health and related quality of life, but it has also empowered especially women to be future health ambassadors in their communities.

### Implications for practice

Several implications may be drawn from the current study. Firstly, the use of local knowledge through the involvement of members of the community together with other stakeholders in planning and making informed decisions on actions has had a significant impact on health related quality of life of members in the community. In addition, the unique role of academicians as facilitators in the process of knowledge mobilization and application of such knowledge to improve health among members of the communities is also an important take home message from this study.

Secondly, participants became certified as health ambassadors after completion of the 12-week physical activity intervention. As health ambassadors, they were encouraged to spread the intervention to other members in their community which participants in this study were willing to do. Finally, participants started also smaller training groups with their friends, families or colleagues at work after the completion of the intervention program, They did not only continue to be physically active but also became role models for their friends and family.

The learnings from the current study is also important when considering designing similar health promotional activities in other socially disadvantaged neighborhoods elsewhere in Sweden or in global context. The authors would like to provide several recommendations to overcome practical barriers when designing similar studies.

- Health promotional activities should not merely be based on participants perceived needs. Rather it should allow the participants to involve in the CBPR process and thereby design for themselves, activities suited to their own needs.
- The role of the health promoter, a representative from the community is considered vital in including members who are otherwise isolated from the rest of the community.
- Multiple training sessions should be offered on different days, and at different times to increase participation and minimize dropouts. Time schedules should be discussed ahead with potential participants.
- Participants should be placed in groups such that they feel comfortable and welcome within their groups. If individuals wish to train only with members of their own gender owing to religious reasons it should be made possible.
- After completion of the intervention, the health promoters or the coach should also mentor participants periodically to boost their motivation.

### Limitations

The study had a small sample size with predominantly females in the group. Despite the small sample size, the study results indicate a positive direction for further large-scale studies in this context. Problems such as isolation including the need to remain indoors owing to sociocultural practices, language barriers and lack of social network leading to physical inactivity is regarded as more common among women in this context compared to men [19]. Hence, the reach out to more women was considered a positive effort to reduce disparities. Furthermore, the results of this study must be interpreted with caution since the intervention may have been too brief to make substantial changes to health behavior. However, participants in this study were inclined towards maintaining positive health behavior and presumed that by being coaches themselves they could also continue training especially since they believed in teaching others through being role models. The research team also intends to perform long-term follow-up measurements, which will further encourage participants to follow routines. The lack of control group in this study also limits the possibility to discuss the causal effect of the intervention on measured parameters.

## Conclusion

The intervention supported by participatory approach presented in this study appears feasible and potentially effective in promoting health in communities in socially deprived regions. In addition, health promotional activities offered as a group activity may improve the effect of the intervention through creating social cohesion. Further research is needed to substantiate the findings from this feasibility study. However, the initial evidence from such interventions can guide future studies using similar models and approaches were community participation is key for both the development of CBPR informed health intervention, as well as evaluation of such programs.

## Data Availability

The datasets generated and analyzed during the current study are not publicly available due copyrights issues and GDPR regulations but are available from the corresponding author on reasonable request.

## Abbreviations

BMI: Body Mass Index
CBPR: Community Based Participatory Research
WHO: World Health Organization
WHOQOL − 100: World Health Organization Quality of Life Survey − 100 items version
WHOQOL-BREF: World Health Organization Quality of Life Survey –Brief Version
